# Adherence to Guidelines for Avoiding Drug Interactions Associated with Warfarin - *A Nationwide Swedish Register Study*

**DOI:** 10.1371/journal.pone.0097388

**Published:** 2014-05-15

**Authors:** Jonatan D. Lindh, Marine L. Andersson, Buster Mannheimer

**Affiliations:** 1 Dept of Laboratory Medicine, Division of Clinical Pharmacology, Karolinska University Hospital, Huddinge, Karolinska Institutet, Stockholm, Sweden; 2 Karolinska Institutet, Department of Clinical Science and Education at Södersjukhuset, Stockholm, Sweden; University of Glasgow, United Kingdom

## Abstract

**Purpose:**

To investigate the extent to which clinicians avoid well-established drug-drug interactions associated with warfarin. We hypothesised that clinicians would avoid combining non-steroidal anti-inflammatory drugs (NSAIDs), tramadol and sulfamethoxazole with warfarin.

**Methods:**

A cross-sectional analysis of nationwide dispensing data was performed in Swedish individuals 18 years or older (n =  7 563 649). Odds ratios of interacting NSAIDs, tramadol and sulfamethoxazole versus respective prevalence of comparator drugs codeine, and ciprofloxacin in patients co-dispensed interacting warfarin versus patients unexposed was calculated.

**Results:**

The odds of receiving an interacting NSAID versus the comparator codeine was markedly lower in patients with warfarin than in the remaining population (adjusted OR 0.21; 95% CI 0.20 – 0.22). Also, the interacting drugs tramadol and sulfamethoxazole were less common among patients dispensed warfarin as compared to the remaining population, although the decrease was much more modest (adjusted OR 0.83; CI 0.80–0.87 and 0.81; CI 0.73 – 0.90).

**Conclusions:**

In conclusion, Swedish doctors in the vast majority of cases refrain from prescribing NSAIDs to patients already on warfarin. Tramadol and sulfamethoxazole are however rarely avoided.

## Introduction

Warfarin, a vitamin K antagonist, offers an effective means of thrombosis prevention. However, it has a narrow therapeutic range [1] which explains its association with frequently occurring serious adverse reactions such as gastrointestinal and cerebral bleeding. Drug-drug interactions are a major risk factor in this regard [2]–[5] especially among the elderly due to a greater exposure to multiple drug use [6].

Non-steroidal anti-inflammatory drugs (NSAIDs) work by inhibiting the synthesis of inflammatory prostaglandins, and may therefore impair the aggregation of thrombocytes [7]. In addition, they damage the gastrointestinal mucosa [8], and increase the sensitivity to warfarin treatment [9], factors all of which contribute to a substantially increased risk of severe bleeding [5], [10], [11]. The interaction between warfarin and NSAIDs is one of the most prevalent clinically relevant drug-drug interactions, and in a large US prescription study 24% of warfarin-treated patients received an NSAID during a two-year follow-up [12], [13].

The analgesic effect of tramadol derives from opioid receptor agonism in combination with norepinephrine and serotonin reuptake inhibition. It increases the sensitivity to warfarin treatment [14]–[16] and inhibits thrombocyte aggregation [17] which may result in an increased risk of gastrointestinal bleeding [18], [19]. For a patient already on warfarin, an alternative analgesic such as codeine should therefore be considered. The prevalence of the warfarin-tramadol interaction is not well-documented, but with more than 100 tramadol prescriptions per 1000 adults annually in Sweden, the interaction is likely to concern a large number of patients [20].

Sulfamethoxazole is a widely used antibiotic. Combined with warfarin, it greatly increases the risk for gastrointestinal bleeding, due to cytochrome P450 enzyme (CYP) 2C9 inhibition [21]–[24]. For warfarin-treated patients in need of treatment of urinary tract infections ciprofloxacin may therefore be a better alternative [25], [26]. Despite the well-documented interaction risk, sulfamethoxazole prescription is common in warfarin-treated patients. For example, a recent US study showed that sulfamethoxazol (in combination with trimethoprim) accounts for 12% of all antibiotic prescription in ambulatory patients taking warfarin, indicating that the interaction could be of significant clinical importance [27].

In Sweden, the SFINX prescribing support software automatically alerts doctors when they are about to co-prescribe a potentially dangerous combination [28]. Guidance is hereby provided on how to handle the specific drug-drug interaction. SFINX is available to approximately 80% of the doctors [29].

Table 1 present in detail information regarding respective interaction provided by SFINX as well as on the labelling of respective study drug according to The Swedish summary of product characteristics [30].

**Table 1:** Rationale for the choice of study drugs.

Labelled indications for groups of drugs according to FASS^1^	Drugs (ATC code^2^)	Role of study drug	Rationale	Recommendations according to SFINX with regard to combining warfarin with respective study drug	Labelling according to FASS^1^ with regard to interactions leading to an increased risk of bleeding
Vitamin-K-epoxide reductase inhibitor The only anticoagulant used in Sweden. Small therapeutic window. Indications include the treatment and prevention of venous thrombosis, to prevent thromboembolism in patients with atrial fibrillation	warfarin (B01AA03)	Interacting drug	Has a narrow therapeutic range and the dose needed for sufficient anticoagulation is close to that which may cause bleeding [1], [44]	NA	NSAIDs^3^ incresase the effect of warfarin pharmacokinectally and pharmacodynamically. Combining with inhibitors of CYP2C9^4^ should be avoided.
Analgesics Sharing indications for treatment of acute moderate pain. NSAID^3^ used for conditions associated with inflammation	NSAID^3^ (MA01A)^5^	Interacting analgesic	Impair the aggregation of thrombocytes [7], damage the gastrointestinal mucosa [8], and increase the sensitivity to warfarin treatment [9] leading to a substantially increased risk of severe bleedings [10], [11].	Co-dispension with warfarin may cause severe bleedings. The combination should be avoided.	May increase the risk of bleeding when used together with warfarin. Close monitoring is warranted.
	tramadol (N02AX02)	Interacting analgesic	Increases the sensitivity to warfarin treatment [14]–[16] and inhibits thrombocyte aggregation [17] which may result in an increased risk of gastrointestinal bleeding [18], [45].	The effect of warfarin may increase when used concomitantly. The combination should be avoided.	May increase the anticoagulant effect of warfarin. Increased monitoring is warranted.
	codeine (R05DA04, N02AA59)	Comparator analgesic	Opiod analgesic. No evidence of interaction with warfarin.	No warnings issued regarding the coadministration with warfarin.	No warnings issued regarding the coadministration with warfarin.
Anti-infectives Sharing indications for treatment of gram-negative bacteria and used for the treatment of urinary tract infections.	Sulfa-methoxazole (J01EE01)	Interacting anti-infective	Substantially increased risk for gastrointestinal bleeding on the basis of cytochrome CYP 2C9^4^ inhibition [21]–[24].	The effect of warfarin and bleeding is markedly increased. The combination should be avoided.	May significantly increase the effect of warfarin. Close monitoring is warranted.
	ciprofloxacin (J01MA02)	Comparator anti-infective	Some data suggest an increase of the anticoagulant effect of warfarin due to an uncertain mechanism although the evidence is conflicting [25], [26], .	May increase the anticoagulant effect of warfarin. Careful monitoring of warfarin is warranted.	May increase the anticoagulant effect of warfarin. Increased monitoring of warfarin is warranted.

1 The Swedish summary of product characteristics.

2 Anatomical Therapeutic Chemical code.

3 Non steroidal anti-inflammatory drugs.

4 Cytochrome P450 enzyme (CYP) 2C9.

5 Include all acetylsalicylic acid drugs beginning with MA01, glucosamine (M01AX05) excluded.

The aim of the current study was to investigate the compliance to guidelines on drug-drug interactions with the potential to cause warfarin induced bleeding. We hypothesized that doctors in Sweden would avoid the combined use of warfarin and interacting drugs.

## Methods

### Ethics statement

This was a database study that included data on the entire Swedish population 18 years or older. Hence we did not interfere with the treatment of these individuals nor in any other way. Since the data was anonymized and none of the individuals were identifiable, the integrity of the individuals was not judged to be violated. This view was also supported by the Regional Ethics Committee in Stockholm, Karolinska Institute, which waived the need for written informed consent from the participants and approved the study as a whole.

### Study design

The study design was a retrospective, cross-sectional analysis of patients being dispensed prescription drugs in Sweden during the period from August 15 to December 15, 2011. The choice of a four-month-study-period was based on the Swedish regulation and experience that most patients on long-term/chronic treatment repeat their drug-dispensing every third to fourth month. We selected all individuals, 18 years or older, that were dispensed any of the drugs presented in Table 1. The cohort was established on data obtained from the Swedish Prescribed Drug Register (SPDR) [31], [32].

### Data source

The Swedish Prescribed Drug Register contains data with unique patient identifiers for all dispensed prescriptions covering the whole population of Sweden. The data collection is administered by the National Corporation of Swedish Pharmacies, a state-owned company responsible for the provision of pharmaceutical services at a nationwide level. Data on all dispensed prescriptions is transferred monthly to the National Board of Health and Welfare. The drugs are classified according to the Anatomical Therapeutic Chemical (ATC) classification system. From this register, we selected all individuals, 18 years and older.

### Variables

We hypothesised that physicians in Sweden would avoid the combined use of warfarin and interacting drugs. If so, the odds ratio between the prevalences of interacting drugs to comparator drug users would be lower among patients co-dispensed warfarin as compared with patients without warfarin, a methodology used previously [33]–[35].



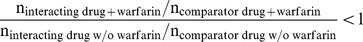



Thus, in the analgesic area, corresponding outcomes were the odds ratio of being prescribed a NSAID (vs codeine) and that of being prescribed tramadol (vs codeine). NSAIDs include acetylsalicylic acid and all drugs with Anatomical Therapeutic Chemical (ATC) codes beginning with M01A, glucosamine (M01AX05) excluded, i.e. celecoxib, dexibuprofen, dexketoprofen, diclofenac, etoricoxib, ibuprofen, indomethacin, ketoprofen, ketorolac, lornoxicam, meloxicam, nabumetone, naproxen, parecoxib, piroxicam, tenoxicam. The grouping of individual NSAIDs into a single exposure category was justified by the fact that COX inhibitors are expected to have similar effects on the thrombocyte function and gastric mucosa [7], [8], [36], and the interaction is generally considered a class-effect of NSAIDs. Consequently, SFINX recommends that prescribers abstain from combining warfarin with any NSAID without differentiating between individual substances [28].

For anti-infectives, the outcome measure was the odds ratio of being prescribed the interacting drug sulfamethoxazole (rather than ciprofloxacin) comparing warfarin users to non-users of warfarin.

In the statistical analysis, factors considered potential effect modifiers were age, gender, number of drugs and medical setting, all treated as categorical variables. The variable age was divided into four groups: young adults, adults, geriatric patients and the oldest (18–44, 45–64, 65–79 and ≥ 80) and the variable number of drugs was divided into three groups (<5, 5–9 and >9). Information on medical setting was based on the variable “Prescribers'working place” in the SPDR and whether the interacting drug was being prescribed from a primary or specialist care unit. Primary care was defined as care provided by health care professionals that often play a role in the local community and act as a first point of consultation for all patients within the health care system. Secondary care was defined as care provided by medical specialists often associated with a hospital such as cardiologists, endocrinologists or other internists.

### Analysis

To study associations between warfarin and the interacting drugs and to control for potential effect modifiers we used multivariate logistic regression. The associations are presented as odds and odds ratios (OR) with 95% confidence intervals (CI). The departure from 1 (no association) is statistically significant at the 5% level, two-tailed, if the 95% CI does not include 1. All statistical calculations were performed in IBM SPSS Statistics 22.0 (SPSS Inc., Chicago, IL, USA).

### Selection of study population

**Figure 1:**
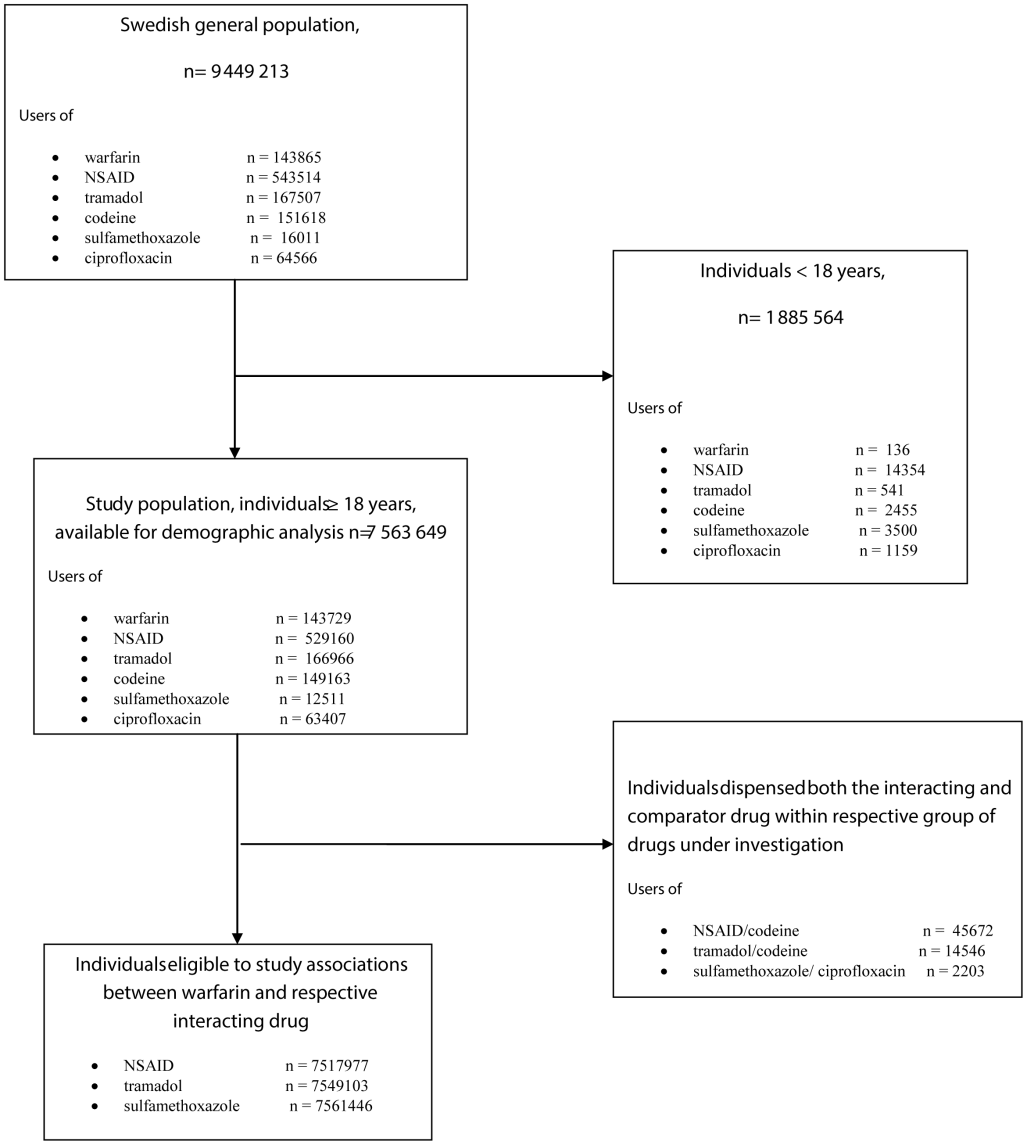
Patient flow chart.

Individuals in the Swedish population 18 years or older (n =  7 563 649) were included in the study [20]. To minimize the possible bias of patients who changed interacting drugs and comparator drugs within the 4-month-study period, associations between different classes of drugs was based on the individuals who had been dispensed no more than one of the drugs in each therapeutic area. Those few individuals who had been dispensed both an interacting drug and a comparator drug were thus excluded (Figure 1).

## Results

The mean age was 49 years, and 51% were women. The prevalence of the use of study drugs in the Swedish study population is given in Table 2 along with corresponding demographics. Warfarin was dispensed to 1.9 percent of the study population (Table 2).

**Table 2:** Prevalence of study drugs used in the adult Swedish population (≥18 years of age) and corresponding demographics, 15^th^ August to 15^th^ December, 2011.

	n	n/1000 individuals	Mean age (SD^1^)	Women (%)	Individuals ≥ 65 years (%)	Percentage of drugs^2^ prescribed from primary care	Mean number of drugs (SD^1^)
warfarin	143729	19	73 (12)	41	81	**65**	7.4 (4.1)
**Warfarin interacting drugs**							
NSAID^3^	529160	70	54 (17)	58	29	**73**	5.3 (4.0)
tramadol	166966	22	59 (17)	58	40	**72**	7.5 (4.7)
sulfamethoxazole	12511	1.7	61 (18)	42	49	**34**	8.8 (5.5)
**Comparator drugs**							
codeine	149163	20	55 (18)	60	33	**61**	6.7 (4.7)
ciprofloxacin	63407	8.4	61 (19)	39	48	**58**	7.2 (5.2)

1 Standard Deviation

2 Defined as a seven-digit Anatomical Therapeutic Chemical (ATC) code. The remaining proportions were prescribed from a specialist care setting.

3 Non steroidal anti-inflammatory drugs include all drugs beginning with M01A, glucosamine (M01AX05) excluded.

**Figure 2:**
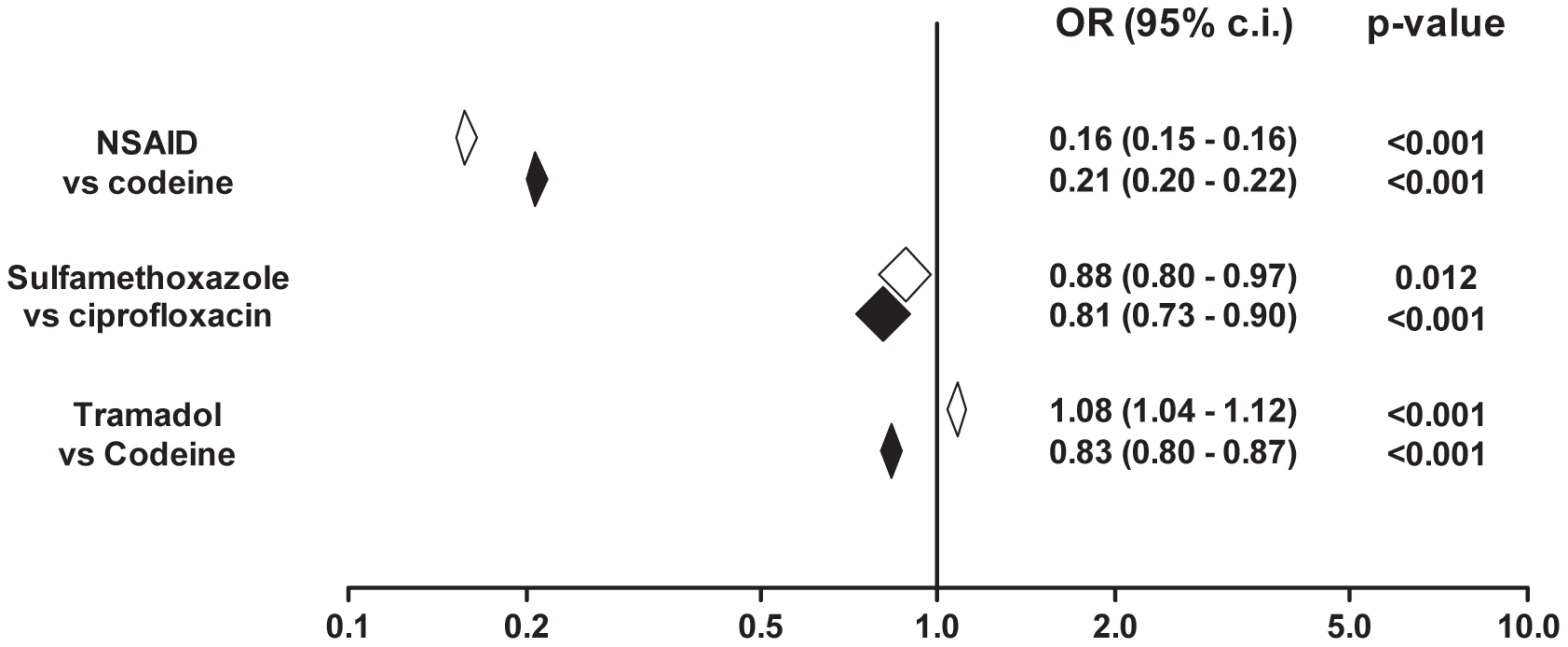
Odds ratios of exposure to an interacting rather than a non-interacting drug in warfarin-treated individuals vs individuals without warfarin. White diamonds represent 95% c.i. of unadjusted ORs, filled diamonds represent ORs adjusted for gender, age, number of drugs and clinical setting.

Table 3 shows the number of individuals in the different groups of patients under comparison. The number of patients dispensed warfarin in combination with NSAID, tramadol and sulfamethoxazole was 4273, 6650 and 464 respectively. Figure 2 shows odds ratios of receiving interacting drugs in patients dispensed warfarin as compared to the remaining population. The odds of receiving an interacting NSAID versus the comparator codeine was markedly lower in patients with warfarin than in the remaining population (adjusted OR 0.21; 95% CI 0.20 – 0.22). The interacting drugs tramadol and sulfamethoxazole were also less common among patients dispensed warfarin as compared to the remaining population (adjusted OR 0.83; CI 0.80 – 0.87 and 0.81; CI 0.73 – 0.90).

**Table 3:** The number of individuals with interactive drugs and comparator drugs among individuals with or without co-dispensed warfarin under the study period 15 August to 15 December, 2011.

	Interactive drugs	Comparator drugs
	NSAID	codeine
warfarin	4273	5516
no warfarin	479215	97975
	sulfamethoxazole	ciprofloxacin
warfarin	464	3114
no warfarin	9844	58090
	tramadol	codeine
warfarin	6650	5472
no warfarin	145770	129145

Table 4 shows the adjusted odds ratios of being dispensed interacting drugs in primary care settings, specialist care settings, individuals < 65 respectively ≥65 years of age, in males and in females. The results in the investigated subgroups were mostly consistent with those for the general population. However some differences were noted. The inverse association between tramadol and warfarin was stronger in primary care (adjusted OR 0.80; CI 0.76–0.84) compared to specialized care (adjusted OR 0.91; CI 0.85–0.98). On the contrary, the inverse association between sulfamethoxazole and warfarin was not evident in primary care patients (adjusted OR 0.87; CI 0.74–1.02) while being pronounced in individuals receiving their prescriptions in a specialized care setting (adjusted OR 0.77; CI 0.67–0.88). Among patients 65 years or older the decreased association between interacting tramadol and sulfamethoxazole respectively and warfarin was stronger as compared to the population as a whole (adjusted OR 0.82; CI 0.78–0.86 and 0.80; CI 0.71–0.90). In younger patients this association was very weak and statistically non-significant (adjusted OR 0.98; CI 0.90–1.06 and 0.90; CI 0.71–1.14).

**Table 4:** Associations between study drugs, in patients with or without dispensed warfarin dispensed in primary care setting, specialist care setting, and in males under the study period 15 August to 15 December, 2011.

Study drugs	Individuals prescribed from primary care, adjusted^1^ odds ratios (95% CI^2^)	Individuals prescribed from specialised care, adjusted^1^ odds ratios (95% CI^2^)	<65 years (n = 2752666), adjusted^1^ odds ratios (95% CI^2^)	≥65 years (n = 1547787), adjusted^1^ odds ratios (95% CI^2^)	Males (n = 1781475), adjusted^3^ odds ratios (95% CI^2^)	Females (n = 2518978), adjusted^3^ odds ratios (95% CI^2^)
Interacting drugs: NSAID^4^	0.20 (0.19 – 0.21)	0.24 (0.22 – 0.26)	0.20 (0.18–0.22)	0.21 (0.20 – 0.23)	0.23 (0.22 – 0.25)	0.19 (0.17–0.20)
Comparator drug: codeine (reference group)						
Interacting drugs: tramadol	0.80 (0.76 – 0.84)	0.91 (0.85 – 0.98)	0.98 (0.90–1.06)	0.82 (0.78– 0.86)	0.86 (0.83 – 0.93)	0.89 (0.84–0.93)
Comparator drug: codeine (reference group)						
Interacting drug: sulfamethoxazole	0.87 (0.74 – 1.02)	0.77 (0.67 – 0.88)	0.90 (0.71–1.14)	0.80 (0.71 – 0.90)	0.79 (0.71– 0.88)	0.82 (0.68–0.99)
Comparator drug: ciprofloxacin (reference group)						

1 Estimates adjusted for gender and age.

2 Confidence Intervals.

3 Estimates adjusted for age and medical setting.

4 Non steroidal anti-inflammatory drugs.

## Discussion

Using complete data on all prescription drugs dispensed during a four-month period in Sweden, we demonstrate that prescribers to a large extent avoid combining warfarin with the potentially interacting NSAIDs. In contrast, sulfamethoxazole and tramadol are rarely avoided in warfarin-exposed individuals, despite the risk of drug-drug interactions.

An obvious explanation for this discrepancy could be that physicians are more aware of the interaction between NSAIDs and warfarin. For example, education on warfarin drug-drug interactions may have put greater emphasis on the NSAIDs, since their use is much more widespread [12], [13] compared to that of tramadol [20] and, in particular, sulfamethoxazole [27]. Also, the NSAIDs' well-known propensity to cause gastrointestinal bleeding *per se* could make the risk of combining them with warfarin more intuitive [7], [8], [37]. On the contrary, tramadol and sulfamethoxazole are prone to cause hemorrhage primarily in patient co-medicating with warfarin [21], [24].

An alternative explanation is that the prescribers are aware of the potential interaction risks, but conscientiously decide to combine warfarin with tramadol or sulfamethoxazole. The documentation of the warfarin-tramadol interaction may be considered too scarce to justify the choice of an alternative analgesic why clinicians instead choose to employ close INR monitoring [14]–[17]. The reason for the quite modest decrease in association between sulfamethoxazole and warfarin may not only represent a lack of knowledge regarding the potential risks of this specific drug-drug interaction, but could also reflect a tendency to prioritize adherence to available guidelines for microbial usage.

The study has important strengths. Most importantly, the large number of prescriptions analysed provided it with sufficient power for very precise effect estimates. In addition, the use of complete data on all drugs dispensed in Sweden during a defined time period means that the results should not be influenced by selection bias regarding prescribers, patients or drug prescriptions.

The study also has some potential weaknesses. Most importantly, the observational design means that the results could have been influenced by various types of bias. Using available register data, we statistically controlled for the influence of age, gender, medical care setting, and number of prescribed drugs, but other potential sources of bias could not be accounted for. For example, we did not have information on treatment indication and although the pairs of interacting and non-interacting drugs were meticulously chosen to represent plausible treatment alternatives, the prescribers' choice could have been influenced by a range of factors other than the risk of warfarin interactions. For example, regarding choice of urinary tract infection antibiotic the antimicrobial resistance pattern may have prevented the prescriber from choosing freely between the two study drugs. In the individual case, a second best option may be to prescribe sulfamethoxazole despite the interaction risk and then employ close monitoring of INR, perhaps in combination with a proactive reduction of the warfarin dose, a strategy that could not be evaluated in the current study. Furthermore, although the data have the advantage of being based on information on dispensed rather than prescribed drugs, it was not possible to ascertain whether the medication was actually consumed. Another uncertainty about the dispensing data relates to the employment of a fixed time window to estimate the use of drug combinations. Although generally regarded valid, applying a time window may be associated with both under- and overestimation of exposure [32]–[34]. An alternative method that sometimes may be associated with less bias is the assessment of concomitantly used drugs at a fixed time point is the “legend time” method, where treatment duration is calculated from the amount of drug dispensed. However, for assessing exposure to drugs used on an “as needed” basis such as analgesics, the validity of this method is questionable being based on an assumption of regular intake [38], [39].

The current study was limited to prescription drugs and over the counter drugs (OTCs) were thus not included. The prevalence of patients on warfarin that were co-dispensed an NSAID (n = 4273) may therefore be underestimated. Although the present study focus on prescribed drugs it cannot be excluded that some physician tells the patient to go and buy an NSAID over-the-counter which is a limitation of the study.

The results from investigating the population as a whole were mostly consistent with the results of the subgroup analyses. However, some differences were noted. The decreased association between interacting tramadol and sulfamethoxazole and warfarin was more pronounced in patients ≥65 years as compared to younger patients. This may indicate an increased effort to avoid warfarin associated interactions in the more vulnerable geriatric population [1]. When comparing the two medical settings the results deviated from the population as a whole as well but in different directions for the two therapeutic areas. The tendency to avoid combining tramadol with warfarin was more pronounced in the primary care as compared to individuals prescribed from specialist care. On the contrary, the decreased association between sulfamethoxazole and warfarin was more marked in the specialist care. The significance of these findings remains unclear.

A few previous studies have investigated prescriber awareness of drug-drug interactions using similar methods [33]–[35]. These studies have indicated that prescribers to some extent do take drug-drug interactions into account when prescribing well-known inhibitors of drug-metabolizing enzymes. However, the extent to which drug-drug interactions have been avoided has generally been small compared to the effective avoidance of NSAID prescription in warfarin-treated patients seen in our study (adjusted OR 0.21). Interestingly, the only previous example of a drug-drug interaction awareness of this magnitude did also involve warfarin. Using prescription data from Ireland, Williams et al. showed that interacting H_2_ blockers are effectively avoided in warfarin-treated patients (OR 0.21 for prescription of an interacting rather than a non-interacting drug) [33]. Inversely, drug interactions potentially resulting in loss of therapeutic effect were very rarely taken into account by Swedish prescribers in a study by Mannheimer et al. [35]. For these interactions, odds ratios were approximately 1, matching those of warfarin-tramadol and warfarin-sulfametoxazole in the present study.

Although the odds receiving an NSAID vs. codeine are about five times lower in patients receiving warfarin, 4273 patients received co-prescriptions of warfarin with NSAIDs. This is likely to represent a considerable clinical problem and is clearly against available guidelines.

The present study indicates a need for improved compliance with drug label recommendations as well as a need for continuous medical education about the basic pharmacology of commonly used drugs. In Sweden, the SFINX prescribing support software provides guidance on how to handle drug-drug interactions, including the ones studied herein [28], [29]. Apparently the Swedish physicians often fail to take advantage of this tool. Prescribers' tendency to override DDI alerts is a well-known problem described from several clinical contexts [40]–[43]. The present study further emphasizes the need to overcome this barrier.

In conclusion, Swedish doctors in the vast majority of cases refrain from prescribing NSAIDs to patients already on warfarin. Tramadol and sulfamethoxazole are however rarely avoided.
